# Motivating factors for individuals aged 40–70 years with type-2 diabetes to engage in physical activity

**DOI:** 10.3389/fpubh.2025.1579255

**Published:** 2025-06-10

**Authors:** Sólfríð A. Skoradal, Sarita Vesturdal Vesturtún, May Britt Skoradal, Annika Helgadóttir Davidsen

**Affiliations:** Faculty of Health Sciences, University of the Faroe Islands, Tórshavn, Faroe Islands

**Keywords:** exercise, physical activity, type-2 diabetes mellitus, motivation, self-determination

## Abstract

**Introduction:**

Type-2 diabetes (T2D) is a chronic disease resulting from inadequate insulin production or impaired insulin function. Globally, T2D poses a significant health burden with various complications. While a healthy lifestyle is key to delaying complications of T2D, sustaining long-term habits such as maintaining a healthy weight, following a proper diet, and engaging in physical activity (PA) remains challenging for many patients. Despite growing awareness and earlier diagnosis of T2D, motivation to adopt and maintain these behaviors is often challenged. Traditionally, motivation has been viewed in terms of strength and quantity, with less focus on its type, quality, and orientation. Self-Determination Theory (SDT) emphasizes the role of three basic psychological needs (BPNs)—autonomy, competence, and relatedness—in fostering intrinsic motivation and well-being. Research suggests that SDT can increase the understanding of lifestyle changes.

**Objectives:**

This study aims to explore the motivational factors influencing PA engagement among individuals with T2D in the Faroe Islands.

**Methods:**

Two semi-structured interviews were conducted with eight people aged 43–69 years, and data were analyzed using thematic analysis.

**Results:**

Findings revealed four main themes: (1) *Fearing consequences*, (2) *Starting exercise*, (3) *Hoping to keep on*, and (4) *Being social*. These themes highlight factors influencing motivation to engage in PA within this group.

**Conclusion:**

The findings provide insight into the motivational drivers among individuals with T2D. Although the sample size is limited, the results suggest areas for further research and potential targets for tailored interventions.

## Introduction

Type-2 diabetes (T2D) is a chronic metabolic disorder characterized by persistent hyperglycaemia resulting from insulin resistance and relative insulin deficiency (1). Hyperglycaemia, or elevated blood sugar, is a common effect of uncontrolled diabetes and can lead to severe damage to many body systems over time, particularly nerves and blood vessels^1^. T2D is closely linked to heredity. A person has a 40% risk of developing T2D if one parent has the disease, and the risk doubles if both parents have it (2, 3). Given the hereditary risk, it is advisable to undergo regular screening for T2D proactively and to adhere to recommendations for physical activity (PA) and a healthy lifestyle in general.

Globally, T2D is a high-burden disease that significantly increases the risk of cardiovascular complications, including heart attack, stroke, and heart failure, as well as other severe outcomes such as blindness, kidney failure, and limb amputation. T2D can thus decrease the quality of life and expected lifespan (4–6). Despite the increasing awareness of T2D, which allows for earlier diagnosis, a challenge is that individuals are often diagnosed late with T2D; at that time, complications can have already caused significant damage to the body. T2D is typically managed through a combination of lifestyle modifications—such as dietary changes, increased PA, and weight loss—and pharmacological interventions, including oral hypoglycaemic agents, subcutaneous medications, and, in some cases, insulin therapy (7). Early detection and timely treatment initiation significantly improve the prevention of disease-related complications and the health-related quality of life.

T2D is closely linked to poor eating habits and a sedentary lifestyle^1^ (8). While T2D cannot be cured, lifestyle changes can slow its progression and even reduce its impact, emphasizing a healthy lifestyle as the maximum informed preventive measure to prevent T2D or delay complications of T2D (see Footnote 1) (9–11). The World Health Organization recommends at least 30 min of moderate PA daily for adults with T2D (see Footnote 1). To improve insulin function, it is recommended to exercise daily or avoid going more than 2 days without it. For optimal glycemic control and overall health, adults with T2D should ideally engage in aerobic and resistance training. Structured lifestyle interventions should include at least 150 min of PA per week (12, 13). Additional recommendations for preventing or slowing T2D progression include maintaining a healthy body weight, avoiding smoking, consuming a balanced diet, and limiting sugar intake (see Footnote 1). Results from a systematic review demonstrated that structured exercise and advice about PA, with or without dietary intervention, effectively reduce glycated hemoglobin levels in T2D patients. These findings highlight the importance of incorporating PA into preventive measures, with supervised training proving more effective than unsupervised programs (14). PA, as defined by the World Health Organization, is *a bodily movement by skeletal muscles that requires energy expenditure* (see Footnote 1). This includes daily activities such as transportation, household tasks, and sports. Incorporating PA into daily routines, such as waking or cycling, can promote health. Both moderate and vigorous intensity levels are beneficial for physical health and overall well-being^2^.

Despite well-known benefits, maintaining a healthy weight, diet, and PA level over the long term remains challenging for individuals with T2D (15). Similar trends are observed in the Faroe Islands, where a 2021 Gallup survey of 517 adults showed a decline in normal weight and an increase in overweight and obesity (16). Recent data from the Faroese Diabetes Association estimates that 3,000 individuals are living with T2D, which represents 5.5% of the Faroese population (17). T2D prevalence appears higher among individuals with short to medium-length education than those with longer education (18). A study of individuals aged 40–74 furthermore found that T2D and prediabetes prevalence increased with age and was higher in men, with slightly higher rates than in other Nordic countries (19).

The majority of PA interventions focus on education, information, and nursing-specific elements, often overlooking key factors such as self-determination, motivation, and social support (15). One study showed that written advice from their general practitioner had no statistically significant impact on metabolic control in T2D patients, but it did increase their medication adherence (20). Adopting a healthier lifestyle often requires significant changes in daily routines, including diet and PA, which can be challenging (21). Self-Determination Theory (SDT) by Ryan and Deci emphasizes autonomous motivation as a driver for lasting behavior change. Traditionally, motivation has been viewed mainly in terms of strength and quantity, with less attention to its type or orientation (22). SDT identifies three basic psychological needs (BPNs)—autonomy, competence, and relatedness—as essential for optimal functioning, growth, and well-being (22, 23).

Over the past 20 years, rehabilitation methods have undergone significant development. Notable progress has been made in medical and occupational rehabilitation, particularly from a social science perspective. However, a comprehensive system that fully captures the complexity of rehabilitation is still lacking, and the definition of rehabilitation remains unclear (24). Contemporary society faces increasing pressure due to an aging adult population, rising prevalence of chronic diseases, and the escalating importance of rehabilitation within healthcare systems (24). Rehabilitation provides substantial benefits for individuals experiencing physical, psychological, social, or cognitive limitations in their daily functioning. To be effective, rehabilitation should be individualized and continuously adapted to align with the individual’s progress (24).

As stated, although lifestyle changes can prevent or minimize the negative consequences of T2D, maintaining healthy habits remains challenging for many. Therefore, this study explores the motivating factors that influence individuals aged 40–70 years with T2D to engage in PA. By gaining insight into personal, social, and environmental drivers of motivation, the study seeks to inform the development of more effective, person-centered interventions to support long-term PA adherence in this population.

## Materials and methods

This qualitative study employed a focus group design to explore motivational factors for PA among individuals with T2D. Our approach was deductive hermeneutic, providing a framework for understanding the layers of meaning in human life, behavior, and culture (25). This approach furthermore focuses on understanding people’s actions (26). Rather than explaining them, as is known in more quantitative science methods, where generalisability is used as a primary focus (27). One of the advantages of using focus groups is the sharing of norms and interactions with each other, as well as interpreting a subject with agreement (26), and that aspect would not be achieved using individual interviews. The study was performed according to general guidelines for research ethics (28) and the University’s code of research ethics.

### Data collection

Using the participant list from a main intervention study called *The Faroese type 2 diabetes training project* (ClinicalTrials.gov ID: NCT06478173), we randomly selected individuals categorized by age, selecting those from the oldest and youngest years. We randomly selected four of the oldest and four of the youngest. The intervention study’s inclusion criteria required participants to have been diagnosed with T2D within the past 10 years and to be between 40 and 70 years of age. We added inclusion criteria regarding sex and geographic diversity, seeking to include both men and women participants from different places in the Faroe Islands. Prior to and in connection with the introduction to the main intervention study, all participants were informed that they might be contacted for an interview study. We received the participant list (including name, address, date of birth, and phone number) from the intervention study. We highlighted individuals in the youngest (40–45 years of age) and oldest (65–70 years of age) age groups. To ensure gender visibility, we assigned distinct markers to women and men. We then reviewed the participants’ geographic locations. For each interview, we selected one woman and one man from the 40–45-year age group and one woman and one man from the 65–70-year age group. We then contacted participants by telephone and planned when and where the interview would occur.

### Interview setting

Both interviews took approximately 75–80 min and were recorded with a dictaphone. They took place in the same neutral office. The first author (SS) was the primary facilitator, and the second author (SV) was permitted to complement the first author during the interviews. Adjustments were made from the first to the second interview, with a more thorough and personalized presentation round by the researchers at the beginning of the interview. This adjustment was made to get the participants to feel more relaxed and comfortable during the interview, compared to the first interview, where we observed possible discomfort in sharing. Before the interviews, participants got and signed written information about the study and the possibility to withdraw at any time without consequences.

### Interview guide

Our opening question was *How was it to get diagnosed with T2D?* followed by *Have you made any changes since your diabetes diagnosis?*. During the interviews, the facilitators narrowed the focus to the subject of PA and motivation. The interview guide was inspired by the SDT, and the questions were aimed toward showing an intrinsic or an extrinsic motivation (see Table 1).

**Table 1:** Interview guide.

Subjects	Area of interest	Questions	Added questions
Introduction	A short resume about the research and information about anonymity and signing the declaration of consent.	Name, age, work/education, hobbies, and possible exercise/sport.	How, what if, what are.
Knowledge about the disease	At this point, we want to know what knowledge the participants have regarding T2D, and what effect it has on their lives.	1: How was it for you to get diagnosed? 2: Have you made any interventions since? If yes, what were they?	Schooling from the general doctor.
Treatment	What are the participants’ insights on T2D treatments?	1: What does the treatment look like that the participants have been offered? 2: How do they experience exercise?	What does a day look like for you?
Motivation	At this point, we want the participants to tell us about their motivation toward the treatment in general, diet and exercise, and forms of exercise.	1: What are your thoughts about the treatment for T2D? 2: What made you register for this intervention? 3: Do you know anybody who has played football?	

### Data analysis

Thematic analysis based on Braun and Clarke was employed (29), going through the six-stage system (see Table 2). The two interviews were analyzed individually during the first phase. Triangulation was undertaken via individual coding, followed by meetings to reconcile codes and possible themes.

**Table 2:** Description of the process.

Phases		Description of the process
1.	Data familiarization	Transcribe data, read, and re-read. Note initial reflections
2.	Generate initial codes	Interesting elements were coded across the entire dataset
3.	Generate initial themes	Organize codes in patterns
4.	Develop and review themes	Construct a thematic map. Discuss themes in the context of the dataset and theory
5.	Final themes	Define themes. Translate quotes. Continuing analysis of findings in the dataset
6.	Report	Present results, discussion, and conclusion

Both interviews were transcribed verbatim. Participants were given pseudonyms and thereby made anonymous, and workplaces and local places were also anonymized.

### Rigor and trustworthiness

To ensure credibility, we used random sampling. Detailed descriptions of the study setting provide transferability. Dependability and confirmability were maintained through an audit trail documenting research decisions, transcripts, and coding (30, 31).

## Results

### Participants

Eight participants participated in the interviews, two men and two women in each focus group. The age range within the group was 43–69 years (see Table 3).

**Table 3:** Focus group participants.

Focus group	Description	Number, gender, and age of participants	Facilitators
1.	Participants with T2D	Four people Females: 69 years old 50 years old Males: 69 years old 43 years old	The first author was the facilitator, and the second author was the observer
2.	Participants with T2D	Four people Females: 69 years old 48 years old Males: 69 years old 43 years old	The first author was the facilitator, and the second author was the observer

### Themes

We found four main themes which will be discussed in the following sections: (1) *Fearing consequences*, (2) *Starting exercise*, (3) *Hoping to keep on*, and (4) *Being social* (see Table 4).

**Table 4:** Final themes and subthemes.

Final themes				
Theme	*Fearing consequences*	Starting exercise	Hoping to keep on	Being social
Sub-theme	*Acquiring knowledge*	Feedback from gadgets and physical test results	Sustaining new habits	Appointments with friends and/or family
	*Personal responsibility*	Feeling good – benefits	Concerns about the stamina	

#### Fearing consequences

*Fearing consequences* was the first central theme we analyzed as a motivational factor for PA. The majority of the participants expressed fear of T2D-related complications, with a shared understanding across both focus groups, although some consequences were new to a few participants.

*‘I know about one person that did not take care of his diabetes and has lost his legs…*. *To begin with he lost his toes and then it just went further on*.’ *(Woman, 48 y/o)*

and

*‘I had a control appointment with an ophthalmologist, because I have T2D, yeah? …. I knew something was wrong, and I went on the defensive…. Then she told me after a while, that I have cataracts.’ (Woman, 48 y/o)*

This woman sees the medical eye lens condition, a cataract, as something that older people get, which shocked her. Although the participants knew that one of the side effects of diabetes is visual impairment, this was seen as a defeat. The sense of defeat may suggest low autonomy, possibly linked to non-contingent self-esteem in the face of failure. Another participant highlighted his own experiences with the connection between a specific food choice and physical symptoms. He explained that he had made some drastic behavioral changes since being diagnosed:

*‘Yes, I now don’t eat white bread at all, that is 100% been cut off, but sometimes I eat pancakes when my daughter makes them, and that’s it. And soda’s been cut off completely.’ (Man, 43 y/o)*

Participants described feared consequences of poor lifestyle choices, which appeared to motivate behavior change. Some withheld Participants described feared consequences of poor lifestyle choices, which appeared to motivate behavior change. During the interview, some withheld information due to work-related experiences, as they were healthcare providers. As stated here:

*‘Mmm, there is a book. But no, I don’t need to lecture anyone.’ (Woman, 69 y/o)*

The facilitator asks the participant to continue to share her knowledge. She deliberates:

*‘The insulin that flows around in the body, you become insulin resistant, yes. And when you eat there is more insulin in the blood, and that can cause you to become tired and feel discomfort, and there is a book called “Eat Yourself Healthy from Type 2 Diabetes.”’ (Woman, 69 y/o)*

The woman with a healthcare background appears to be more autonomous, given her knowledge, it does not appear to affect her self-esteem as she seeks to manage her T2D, and it does not seem to be an emotional defeat. Quotes from the *Fearing consequences* theme illustrate a clear link between awareness of T2D complications and participants’ food and lifestyle choices. Quotes from the *Fearing consequences* theme illustrate a clear link between awareness of T2D complications and participants’ food and lifestyle choices. Fear appears to be a key motivator, though emotional responses varied, suggesting different types and qualities of motivation.

##### Acquiring knowledge

One subtheme to *Fearing consequences* is *Acquiring knowledge.* It was a common understanding in both groups that having T2D requires some changes in diet and the level of PA. During the interviews, participants shared knowledge from various sources—mainly self-sought, though some came from healthcare providers. Those working in healthcare often emphasized their fears based on professional experience:

*‘I think that with my job as a nurse, I see the consequences of diabetes, and that gives like a kick in the butt.’ (Woman, 50 y/o)*

In addition, we believe that their professional health knowledge may be a reason to focus on a healthy lifestyle and prioritize being physically active:

*‘The well-being of being active, I think. So, you won’t end up like cooked spaghetti’…. ‘Because the older you get, the faster your muscles and bones weaken’. (Woman, 50 y/o)*

Within this subtheme, healthcare practitioners demonstrate broader knowledge about T2D and the body, which appears to foster more autonomous, intrinsically driven motivation.

##### Personal responsibility

Another subtheme under the central theme *Fearing consequences* is *Personal responsibility.* Following a T2D diagnosis, participants’ sense of responsibility shifted, leading all to make behavioral changes. While their paths varied, for some, fear was a primary driver—as one participant clearly expressed:

‘*I said to myself OKAY, do I want to lose my foot, get blind or just eat less sugar. When you think of it like that, the choice is much easier to make.’ (Man, 43 y/o)*

This quote can give a presumption of the man being introjected, regulated, with a sense of internalized nagging for making the right choice. Some statements reflected empowerment through gaining knowledge about self-care and treatment, enabling participants to take responsibility and act to delay potential complications:

*‘will, with all my power, do what it takes, to not end up in that group’*^3^ *… ‘YES, if I can do anything to improve my own health… then…yes!’ (Woman, 48 y/o)*

and

*‘I hate being physically active… but now I intend to do what is right’ (Man, 69 y/o)*

Expressions of dislike for PA reflected a clear sense of amotivation. In contrast, other statements indicated identified regulation, with participants acknowledging the need for change as part of *Personal responsibility*. The theme of self-responsibility emerged through comments about having to make changes themselves, often framed as a choice between serious complications and adopting healthier habits −*lose my foot, get blind, or just eat less sugar*. While it is unclear where participants learned they must take *Personal responsibility*, they appeared to possess the necessary knowledge but expressed uncertainty about acting on it. Statements like *I intend to do what is right* suggest awareness without clear motivation. Their participation in the intervention study appeared to reflect a desire to find or strengthen motivation, signaling a deliberate step toward taking responsibility for their health.

#### Starting to exercise

The second main theme, *Starting exercise*, highlights the diverse experiences of participants in adopting PA. While some had successfully integrated exercise into their daily routine, others were struggling to find enjoyment and intrinsic motivation. The variation is clearly illustrated by contrasting accounts from two participants:

*‘I hate being physically active.’ (Man, 69 y/o)*

and

*‘…I have always done some kind of sports or physical activity.’ (Woman, 50 y/o)*

The first quote shows an example of amotivation, while the second shows intrinsic motivation. One stated when she retired from work, she needed some kind of regular PA to attend to:

*‘I am now retired, and started going to gymnastics, there’s a physiotherapist. It is VERY good. You do exercises for the entire body’ (Woman, 69 y/o)*

The quotes reveal clear differences in participants’ enjoyment and experience with exercising, which influenced how they spoke about it. For some, it was a long-standing habit and a natural part of daily life, reflecting high levels of autonomy, competence, and relatedness. For others, it felt more like a struggle, likely affecting their motivation to engage in PA.

##### Feedback from gadgets and physical test results

Several participants cited feedback—both from high-tech devices and visible weight loss—as a key motivator for lifestyle changes, supporting the idea that positive feedback can enhance intrinsic motivation. One participant, for example, found tracking progress with a data-collecting bathroom scale highly motivating:

*‘I have lost weight…. I bought this bathroom scale called* [name of product]*. And it is connected to the phone and the data goes directly on the phone. I started using it in 2022, and then it shows a graph. And that is motivating to see.’ (Man, 69 y/o)*

One participant shared that weight loss motivated her to continue being physically active and eventually turn it into a regular exercise routine:

*‘It’s been going steadily downward with weight loss. Yes, I became more motivated, when I saw it was going the right way, then I started to go for walks. The walks became longer and longer. Now I’m exercising, I have always exercised, but now it’s much more.’ (Woman, 48 y/o)*

When discussing PA, participants commonly believed that it lowers blood sugar levels. This assumption prompted us to explore where they had acquired this knowledge. When asked if they had experienced this personally, one participant shared that they use a blood sugar monitor to track their condition and had specifically investigated the effect of exercise on their levels:

*‘Yes, I have also tried measuring it. And I have measured it and done some heavy exercises and measured it afterward. And then it* [blood sugar] *has gone down. Physical exercises, that is. I also have a dog that I must walk, and that helps as well.’ (Woman, 69 y/o)*

These statements highlight the need for feedback to demonstrate the impact of their actions. Motivation appears to be driven by positive results, which inspire the desire to stay active and live healthier. However, there are varying needs for information—some participants have found methods to stay informed, while others are still seeking a motivational factor to maintain PA.

##### Feeling good – benefits

For some of the participants, the motivation to continue stemmed from the ‘feel good’ feeling they had previously experienced after engaging in PA. However, they struggled to put into words the specific benefits they gained from these experiences:

*‘But I just feel like, I feel much better… I exercise pretty much also…’ (Woman, 50 y/o)*

Knowing the ‘feel-good’ effect after PA is a motivation, and gives a ‘push’ even if the working day was hard and feeling tired:

*‘One feels like, when I’ve been, yes, I usually go down to the running track. And walk around and run a bit. Feeling much better when coming home. And also, now with football. Especially feeling good.’ (Woman, 69 y/o)*

Some specific physical benefits were stated, such as having a better night sleep and further what we believe is to preserve a robust body:

*‘My night sleep is better when I’m being physically active’ (Man, 69 y/o)*

and

*‘The well-being of being active, I think. So, you won’t end up like cooked spaghetti’ (Woman, 50 y/o)*

While it may be simplistic to claim that a better night sleep only benefits the physical body, participants noted that PA also improves mental well-being. The difficulty in articulating these benefits may be reflected in the words of philosopher Søren Kierkegaard:

*‘Kierkegaard says: “if you walk, it will all be fine.” Because when you walk, you get, then your head it is cleaning up, and, and that gives a well-being, that well-being, the mental well-being gets better when you walk, says Kierkegaard’ (Man, 69 y/o)*

Although participants find it difficult to define the benefits of PA, they describe a familiar ‘feel-good’ sensation that benefits both body and mind. They do not distinguish between exercise and PA, possibly viewing a walk as mentally beneficial and exercise as physically so. However, they do not explicitly differentiate the two or discuss whether the body and mind can be considered separate. The ‘feel-good’ feeling may contribute to satisfaction in BPN, a key element of SDT.

#### Hoping to keep on

The third main theme focused on participants’ hope to sustain their behavioral changes. One participant expressed both a desire and concern about maintaining these changes, noting that past experiences had given him the intuition that he might easily revert to old patterns:

*‘I was on Ozempic…….and the weight was decreasing, and I was doing well…. then the pharmacy was out of stock with Ozempic……. And then I thought: “never mind” and started eating again…this is not an excuse, I know, but I know where the problem is, just saying. And then it just went downhill again, you know.’ (Man, 69 y/o)*

He further explained that participating in the main intervention study (from which the interview participants were recruited) had impacted both his finances and time, and he was committed to giving it a serious effort, hoping to develop intrinsic motivation. It appeared he viewed managing his diabetes as a personal project:

*‘I am using 10 hours a week on this project, yeah? That’s 40 hours a month. That’s 2000-3000 DKK I am using a month, so that you can write a PhD or masters, it’s not anything in it for me, aside from the fact that I might live longer. But for me, I have to use 200 hours on your project, which then also becomes my project, ha.’ (Man, 69 y/o)*

A key strategy for building and maintaining a healthier lifestyle was setting small, achievable goals while avoiding overly strict rules. This approach was seen as a way to make the process more manageable and reduce the risk of failure:

*‘When I started, I just set one goal; I didn’t say that I would never eat sugar again. Because I know that if I did, it wouldn’t have worked.’ (woman, 48 y/o)*

These statements reflect the challenges of maintaining a new lifestyle, alongside concerns and strategies for success. A ‘small steps’ approach and creating a personal plan were seen as key, indicating identified regulation. This subtheme highlights how participants view their efforts as both a personal and financial investment. While acknowledging that not everyone can afford participation, it emphasized the personal and the value such engagement brings to the broader goals of the project.

##### Sustaining new habits

‘Sustaining new habits’ was a general topic among all participants; some of them had made major changes, while others had taken on small goals and added on during the process. A crucial factor was whether the change was enjoyable and fitted into daily life:

*‘Well yeah, I think it’s good to begin the day with swimming, you get freshened up. Nice to shower there, as you would do anyways, so I like it a lot.’ (Man, 69 y/o)*

Participants also expressed the need for friendly reminders from friends or family to help maintain their new habits and stay on track. One said:

*‘The kids give me a push, and they are doing a good job to get me going.’ (Man, 43 y/o)*

The participants highlighted the challenge of following a standard formula, emphasizing that one size does not fit all. This underscores the need for individualized approaches and possibly expanding educational support to accommodate diverse needs:

*‘No, I don’t believe there are two people who have 100 percent the same recipe. You have to invent yours, and I’ll invent mine.’ (Man, 69 y/o)*

The participants explained how different interventions are necessary because people are not the same. Furthermore, it is essential that the intervention fits into their daily lives and that it can be enjoyable or have an enjoyable aspect to it.

##### Concerns about the stamina

Two participants had experienced serious complications often associated with diabetes—one had a stroke and the other a heart attack—both followed by rehabilitation. These events brought significant emotional strain and concern about the future. For one in particular, the fear of recurrence served as a powerful motivator to maintain a healthier lifestyle, as reflected in their determined statements during the discussion:

*‘After the cardiac attack, the doctors told me that it is very likely that it will happen again. Because my veins are in such bad condition.’ (Man, 43 y/o)*

When asked if this message motivates him even more to be exercise regularly, he answered ‘yes’.

Both have been participating in a supervised training program related to their attacks. A statement in relation to difficulties in keeping on training after the program did occur:

*‘No. Yes, I also received rehabilitation after the blood clot, I did. As I remember, I had it three times a week’ …‘Yes, I believe it lasted for three months if I remember correctly. Then I started going to the gym after that to have something to keep it somewhat up.’ (Man, 43 y/o)*

One of them found it effortless to visit the swimming area every day. He enjoyed starting his day with a refreshing swim, which also prepared him for work:

*‘That’s why I think it’s really good, for me, to swim. There I get a bit of physical movement. I vary my swimming. I can crawl and do breaststroke. I can’t do more than that.’ (Man, 69 y/o)*

When asked whether they find it difficult to maintain training on their own, participants responded with a clear *yes*. They expressed a need for encouragement—*a push* from others. Starting a rehabilitation program provides external regulation through guidance, but continuing the program can gradually shift toward a more internalized, introjected motivation. While they are not yet intrinsically motivated, they recognize the importance of PA and rely on external support to stay on track.

#### Being social

Being physically active or exercising was especially meaningful when done with others. Participants shared various examples of group activities and unanimously emphasized the importance of the social aspect in staying motivated:

*‘I am now retired, and started going to gymnastics, there’s a physiotherapist. It is VERY good. You do exercises for the entire body…. and it is a woman and a men’s team …. afterwards we eat and talk.’ (Woman, 69 y/o)*

and

*‘Some of us just want to have fun, and, yes, just being active and having fun. That’s the good part.’ (Man, 69 y/o)*

In addition, participants highlighted the importance of having others plan the program and provide encouragement, as this support helped them stay committed and continue their efforts:

*‘Well… yeah, when I do something, it’s fitness and things like that. Yes. Like group classes. And then it’s group classes, and I feel, yeah. Yes, I actually feel like I get a bit more out of it when I attend group classes. Because then someone else, well, pushes you. You just follow along; someone else tells you what to do.’*

When discussing their motivation and strategies for making changes, several factors emerged, but the social aspect stood out as particularly significant for staying active. Whether it involved joining a team or scheduling activities with friends or family, a crucial element was committing to someone other than themselves. Based on the participants’ statements, their motivation appears to be strongly tied to the social aspect of PA, highlighting the importance of being in a supportive group setting. This reflects the BPN for relatedness as described in the SDT. Having an instructor, trainer, or someone knowledgeable about exercise to guide and encourage them is also important.

##### Appointments with friends and/or family

Having scheduled training sessions with someone, either a friend or a team session, helps with the discipline to show up. It was expressed like this during the interviews:

*‘Me and my friend are going to the gym together 3 times a week……. we text each other: “what time today?” and then one of us says a time, and then we meet up.’ (Man, 43 y/o)*

In general, participants’ statements did not distinguish between PA and exercise when expressing their activities in daily living. But it was clear that they were more likely to attend when there was a sense of relatedness:

*‘I have played a lot, or my son and I have been playing golf. At the beginning I did it for exercise, but it’s really nice. So, we have played a lot…. around 15-18 km. And that’s good.’ (Man, 69 y/o)*

and

*‘If someone calls and asks: “shall we go for a walk,” then I’ll go. I don’t feel like going myself’ (Woman, 69 y/o)*

It appears that participating in a PA within a social context has a positive impact on motivation. This might be because they feel accountable to others if they fail to show up for an arrangement. Additionally, it suggests that they are more inclined to be active with others because it is enjoyable and creates satisfaction in some parts of BPN.

## Discussion

This study explored motivational factors influencing PA among individuals with T2D through two semi-structured focus groups with eight participants. Using thematic analysis, we identified four main themes: (1) *Fearing consequences*, (2) *Starting to exercise*, (3) *Hoping to keep on*, and (4) *Being social*. These themes offer insights into how these factors relate to satisfying BPNs.

The first finding was the theme of *Fearing consequences*, which catalyzed lifestyle changes such as improved diet and increased PA. According to the SDT, fear is a form of controlled, introjected motivation. Over time, however, this can shift toward a more internally regulated motivation, particularly as individuals associate PA with positive experiences (e.g., ‘feel-good’ sensations) after exercise. This potential shift from extrinsic to intrinsic motivation is central to SDT, highlighting the roles of autonomy, competence, and relatedness in sustaining motivation. For example, one participant described feeling exceptionally positive and more self-determined after football training. While fear initially drove some participants, their motivation evolved as they gained confidence and enjoyment in PA. Others doubted their competence, such as feeling unskilled in football, which impacted their motivation. Still, developing a sense of mastery is key for sustaining long-term behavior change. As SDT suggests, intrinsic motivation is more likely to emerge when individuals feel capable and in control of their actions.

The theme of *being social* also emerged as a key motivational factor, with many participants emphasizing that having fun while engaging in activities significantly impacted their persistence, even if their level of competence was not ideal. This theme was intertwined with *starting to exercise* and *hoping to keep on*, showing that participants’ motivation to starting and maintaining PA appeared to be strongly tied to the external social aspect, suggesting that social connections, enjoyment, and relatedness can enhance motivation, in line with SDT’s emphasis on the basic needs of social support and connections.

Although extrinsic motivational factors were more commonly expressed in the interviews, participants may have been reluctant to disclose intrinsic drivers in a group setting, limiting insights into internal motivation. As Umpierre et al. (14) observe, supervised exercise often leads to better outcomes than unsupervised exercise. This was reflected in the study: participants who had faced serious health events (e.g., blood clots) struggled to maintain routines but found motivation through social support, engaging family, or joining group-based activities like football. Others reported greater benefits when exercising with a trainer or in a structured group setting. These findings align with SDT, which emphasizes the importance of social support in maintaining long-term motivation.

Research indicates that distributing pamphlets to this patient group has not proven effective in sustaining better lifestyle habits, such as maintaining regular PA (32). Furthermore, data from our study indicate that participants need others to take responsibility for planning their exercise program or for participating alongside others. There is a need for external encouragement and organization. Research by Arambepola et al. (33) on text messaging interventions highlights the potential for brief, external prompts to influence the maintenance of health behaviors, such as improving health outcome control in individuals with T2D. This aligns with the broader theme of extrinsic motivators playing a role in behavior change, especially when combined with social support and a sense of autonomy. Participants were selected based on gender and age from the participant list of the main intervention study, indicating that they had voluntarily chosen to join the study focused on the effects of a PA intervention on T2D. Therefore, it can be assumed that they were motivated to exercise before starting. This means that our study excludes individuals who potentially find it difficult to identify factors that contribute to maintaining PA or do not wish to participate in the intervention group, which involved playing football fitness three times a week. We had initially intended to conduct the interviews before the participants were randomized into either the control or intervention group, to minimize the potential influence of group allocation on their responses. However, this was not realistic due to the intervention study’s timeline. We therefore decided to proceed with the interviews as planned, even though participants from the intervention group had already attended a few football training sessions. These sessions took place early in the program, meaning they were not fully accustomed to the football training format. This circumstance did have some influence on the group dynamics. They tended to refer to certain shared experiences as obvious or implied understandings stemming from their involvement in the training sessions. Reflections on the first interview were that persons in the control group tended to be quieter, but the same tendency was not in the second interview.

In summary, our study suggests that motivation to maintain healthy behaviors is multifaceted, shaped by both internal and external regulation. Social support and a sense of competence were central to sustaining long-term change. This aligns with SDT, which highlights autonomy, competence, and relatedness as key to lasting motivation. Only one participant expressed clear intrinsic motivation, linking it to a lifelong habit of exercise shared within her family—a likely factor in her sustained PA.

## Conclusion and implications of the study

This study provides insight into the motivational factors influencing PA among individuals living with T2D in the Faroe Islands. Thematic analysis revealed four central themes: *Fearing consequences*, *Starting exercise*, *Hoping to keep on*, and *Being social*. These findings illustrate the complexity of motivation, ranging from controlled forms such as a fear of complications to more autonomous motivations linked to enjoyment and social connection.

Although the sample size was limited, the results align with Self-Determination Theory (SDT), suggesting that motivation can shift over time from external regulation toward more internalized forms. Satisfaction of BPNs—autonomy, competence, and relatedness—appears critical for supporting sustained engagement in PA.

Clinical implications include the need for tailored interventions that not only provide information and structure but also foster internal motivation. Social support—through group activities or family involvement—can strengthen relatedness and enhance adherence to PA and a healthy lifestyle. Furthermore, integrating SDT-informed strategies in healthcare settings may improve the quality of motivation by empowering individuals to feel competent, autonomous, and socially supported in their efforts to maintain a healthy lifestyle. Structured rehabilitation programs that feature gradual transitions to self-management can be particularly beneficial for maintaining long-term behavioral change.

### Strengths and limitations

The strengths of this study’s qualitative approach lie in the valuable insights offered by individuals diagnosed with T2D. However, some limitations must be acknowledged. The participants’ prior enrolment in a larger project may have introduced bias, as they were likely already motivated to initiate change or explore new approaches related to T2D management. Additionally, the small sample size restricts the generalisability of the findings.

Data saturation was reached when we observed no new insights or variations emerging in the themes. As Halkier (26) notes, saturation in focus groups is less about participant numbers and more about when meaning-saturated patterns repeat. In both interviews, similar themes and perspectives appeared. PA was rarely mentioned spontaneously and only arose when we introduced terms like ‘physical activity’ and ‘motivation’—a key analytical finding. After around 80 min, discussions began to repeat or shift off-topic, and further probing did not generate new insights. We therefore concluded that thematic and analytical saturation had been achieved. However, we only had two focus groups, and we could have gained additional, more diverse insights if we had conducted the study with more focus groups. As mentioned, we adjusted our interview style between the two interviews to try to get a more relaxed atmosphere. This may have introduced a bias, as participants in the second focus group might have shared more sensitive information, thereby skewing the thematic balance. Another factor that may have influenced the interviews is that two participants were diagnosed with pre-diabetes. Instead of T2D, one individual in each group. This reduced the homogeneity of the groups and potentially weakened the study’s credibility. To address this limitation, future research should include larger and more diverse samples that are not affiliated with any ongoing research projects to explore whether the motivational themes identified in this study are generalizable across different populations. Longitudinal studies could provide insight into how motivation evolves over time, especially the shift from externally regulated to more autonomous forms of motivation. Additionally, integrating SDT-based quantitative assessments could help link specific motivational profiles to PA behaviors and outcomes. Further investigation into the role of social support and feedback tools—such as wearables or group-based programs—may enhance understanding of how to sustain long-term lifestyle changes.

## Data Availability

The raw data supporting the conclusions of this article will be made available by the authors, without undue reservation.

## Footnotes

^1^ https://www.who.int/news-room/fact-sheets/detail/diabetes#:~:text=Type%202%20diabetes%20affects

^2^ https://www.who.int/news-room/fact-sheets/detail/physical-activity

^3^Group of patients with T2D.
